# Analysis of Patient Income in the 5 Years Following a Fracture Treated Surgically

**DOI:** 10.1001/jamanetworkopen.2020.34898

**Published:** 2021-02-08

**Authors:** Nathan N. O’Hara, Gerard P. Slobogean, Niek S. Klazinga, Dionne S. Kringos

**Affiliations:** 1 Department of Orthopaedics, University of Maryland School of Medicine, Baltimore; 2 Amsterdam Public Health Research Institute, Department of Public and Occupational Health, Amsterdam UMC, University of Amsterdam, Amsterdam, the Netherlands

## Abstract

**Importance:**

Orthopedic injury is assumed to bear negative socioeconomic consequences. However, the magnitude and duration of a fracture’s impact on patient income and social insurance benefits remain poorly quantified.

**Objective:**

To characterize the association between orthopedic injury and patient income using state tax records.

**Design, Setting, and Participants:**

This cohort study included adult patients surgically treated for an orthopedic fracture at a US academic trauma center from January 2003 through December 2014. Hospital data were linked to individual-level state tax records using a difference-in-differences analysis performed from November 2019 through August 2020. The control group comprised of data resampled from fracture patients at least 6 years prior to injury.

**Exposures:**

An operatively treated fracture of the appendicular skeleton.

**Main Outcomes and Measures:**

The primary outcome was individual annual earnings up to 5 years postinjury. Secondary outcomes included annual household income and US Social Security benefits for 5 years postinjury and catastrophic wage loss within 2 years of injury.

**Results:**

A total of 9997 fracture patients (mean [SD] age, 44.6 [18.9] years; 6725 [67.3%] men) to 34 570 prefracture control participants (mean [SD] age, 40.0 [20.5] years; 21 666 [62.7%] men). The median (interquartile range) of preinjury wage earnings was $16 847 ($0 to $52 221). The mean annual decline in individual earnings during the 5 years following injury was $9865 (95% CI, −$10 686 to −$8862). Annual household income declined by $5259 (95% CI, −$6337 to −$4181) over the same period. A fracture was associated with a $206 (95% CI, $147 to $265) mean annual increase in Social Security benefits in the 5 years after injury. An injury increased the risk of catastrophic wage loss by 11.6% (95% CI, 10.5% to 12.7%). Substantial relative income loss was observed in patients with preinjury earnings in the top 3 quartiles, but changes in income were negligible for patients with preinjury earnings in the bottom quartile (19%; 95% CI, −4% to 48%).

**Conclusions and Relevance:**

In this cohort study of patients surgically treated for an orthopedic fracture at a US academic trauma center, fractures were associated with substantial individual and household income loss up to 5 years after injury, and 1 in 5 patients sustained catastrophic income loss in the 2 years after fracture. Gains in Social Security benefits offset less than 10% of annual income losses.

## Key Points

**Question:**

How is orthopedic injury associated with changes in patient income?

**Findings:**

In this cohort study at an urban, academic medical center in Maryland using state tax records, a fracture treated surgically was associated with a $9865 loss in annual individual earnings, a $5259 loss in annual household income, and a $206 increase in annual Social Security benefits over 5 years following injury.

**Meaning:**

The gains in Social Security benefits associated with orthopedic injury do not offset the loss in income for the patient and their household.

## Teaser

This cohort study of patients treated for fractures examines state tax records to assess changes in patient income following injury.

## Introduction

Orthopedic injury often has negative socioeconomic consequences for the patient.^1^ A 2020 meta-analysis^2^ suggests that patients remain absent from work an average of 102 days after their fracture, and one-third of fracture patients do not return to work within 12 months of injury. The magnitude and duration of a fracture’s impact on patient income remain unclear. Prior research has not accounted for the correlation between the injury and preinjury financial conditions, and rarely distinguishes between individual and household effects.^3^

Negative wealth shocks and job displacement have health consequences across the socioeconomic spectrum.^4,5^ Further, adverse health events can affect the patient’s economic well-being.^6,7^ Protection against the financial effects of injury is a major rationale for health and social welfare policy. However, little is known about the impact of sudden health events, such as a fracture, on the magnitude and duration of income loss.

The objectives of this study were to (1) determine the magnitude and duration of a fracture’s association with the incomes of patients and their households; (2) assess if the available social insurance mechanisms offset this income deficit; and (3) identify policy-relevant subgroups particularly at risk of income loss after injury.

## Methods

### Study Design

We performed a retrospective cohort study with nonequivalent controls using a difference-in-differences analysis that linked data from a single academic trauma center in Maryland to state tax records. The study was approved by the University of Maryland institutional review board under a waiver of informed consent. Data linkage was performed in a secure data enclave by the Comptroller of Maryland data warehouse team. Individual tax records remained masked to research team members not affiliated with the Comptroller of Maryland. This study followed the Strengthening the Reporting of Observational Studies in Epidemiology (STROBE) reporting guideline.

### Study Participants

We identified all adult patients with an appendicular fracture treated surgically, based on *Current Procedural Terminology* (CPT) codes, at a state-legislated primary adult resource center for trauma from January 1, 2003, through December 31, 2016. Social Security numbers were obtained through hospital billing records and individually linked to state tax records from 2000 through 2018. We excluded patients without recorded Social Security numbers, patients with a subsequent fracture admission during the study period, and patients with a severe concomitant traumatic brain injury or spinal cord injury (defined as an Abbreviated Injury Scale score of 5 or greater).^8,9^

### Main Exposure

The exposure was a surgically treated fracture of the appendicular skeleton. The primary assumption of a difference-in-differences analysis is that the trends observed in the controls are a valid counterfactual for what would have occurred in the exposure group if the fracture was not sustained.^10^ Fractures are not randomly distributed.^11,12^ Certain risk-taking behaviors and environmental hazards place specific subpopulations at an increased fracture risk. To comply with the parallel-trends assumption, this study’s control group was developed using exposed patients in the years before their index exposure. The tax year in which the fracture occurred for patients in the exposed group was used to anchor the covariate construction of the control group. For example, the tax data of patients who suffered a fracture in 2003 were compared with the 2003 tax data of patients who sustained a fracture between 2009 through 2016. Time zero was defined as the calendar year of injury for the exposure group.

### Study Outcomes

Tax-reported income was evaluated up to 5 years after injury. The primary outcome was individual earnings, obtained from pretax wages and salary reported on W-2 tax forms. Secondary outcomes included household income, Social Security benefits, and catastrophic wage loss. Household income was estimated using the federal adjusted gross income of the patient’s household and includes wage earnings, Social Security benefits, unemployment compensation, workers’ compensation, disability insurance, and most legal settlements. Social Security benefits were reported at the individual level and included Supplementary Security Income, Disability Insurance, and Social Security Retirement Income. Catastrophic wage loss was defined as a ratio of less than 0.5 when dividing the sum of the mean wage earnings in the year of the injury plus 2 years postinjury by the sum of the mean wage earnings in the 2 years prior to injury.^13,14^ We adjusted incomes for inflation using the Consumer Price Index and reported in 2018 US dollars. We recoded the earnings of individuals with negative incomes or nonfilers to zero, consistent with the methods previously used by Chetty et al.^15,16,17^

### Covariates

Demographic and injury characteristics were obtained from patient medical records and tax data. Demographic data included age, sex, race/ethnicity, and type of health insurance. Clinical data included the mechanism of injury, Abbreviated Injury Scale scores for all body regions, location of the fracture, and the number of surgical procedures associated with the index injury. Comorbidity data included tobacco use, hypertension, depression, diabetes, alcohol use, and drug use. Each individual was assigned an Area Deprivation Index (ADI) value based on their tax return address in the year before time zero. ADI measures neighborhood deprivation based on income, education, employment, and housing quality indicators from the 2011-2015 American Community Survey.^18,19^

### Statistical Analysis

Data analysis was conducted between November 19, 2019, and August 13, 2020. We used an equivalence test to evaluate the parallel-trends assumption for individual income, household income, and Social Security benefits.^20^ Prefracture income trends were similar between the fracture and control groups (eTable 1 in the Supplement). We detected a difference in the prefracture Social Security trends, yet the equivalence margin was within $200.

We fit mixed-effects regression models for patient income as a function of exposure group status, the preinjury or postinjury period, and their interaction. The interaction term is the difference-in-differences estimate. Two exogenous effects were of concern during the estimation. First, after years of real wage growth in the early 2000s, the US has experienced a stagnation in wages, most pronounced in low- and middle-income earners, from the mid-2000s until present.^21^ Second, rapid real wage growth experienced by individuals in their 20s and 30s attenuates in one’s 40s and 50s.^22^ We included a dummy variable for the calendar year and conditioned our estimates based on the patient’s age at time zero to account for these exogenous forces. We assumed that other patient factors associated with injuries and patient income would be similar over the duration of the study. All effect estimates were reported as average absolute differences and relative effects from the year of the fracture to 1 to 5 years after injury, as calculated using longitudinal models. For the estimates of relative effects, outcomes were transformed with a natural log plus 1 for modeling and then reported after subtracting the exponentiated coefficient from 1.^23^ In all models, standard errors were clustered by individual to account for the resampling of the controls. The association between a fracture and catastrophic wage loss was estimated using logistic regression.

We estimated subgroup differences in individual earnings and Social Security benefits using a difference-in-difference-in-differences model. Subgroups that were analyzed included the wage income quartile in the year prior to time zero, patient age (<65 years vs ≥65 years), sex, race (White vs racial minority group), fracture severity (open vs closed fracture), fracture region (proximal vs distal), health insurance status, and neighborhood deprivation, measured as the ADI quartile in the year before time zero. Further, the primary income models were replicated within subsets based on the anatomical location of the fractures, including humerus, radius or ulna, femur, tibia, pelvis, hand, or foot. We did not adjust for multiple testing, and all subgroup estimates should be considered exploratory. Estimates are reported as multiplicative effects and can be interpreted as the increase or decrease in income relative to counterfactual gains in earnings after time zero.

Missing covariate data were imputed using multiple imputations.^24^ We performed a sensitivity analysis for the catastrophic wage loss model with alternative definitions of catastrophic wage loss, ranging from 25% to 75%, and varying the time horizon from 2 to 5 years postinjury. A 2-sided *P* value < .05 was considered statistically significant for models. All statistical analyses were performed using R version 3.6.1 (R Project for Statistical Computing).

## Results

### Sample Characteristics

In total, we compared 9997 patients with fractures (mean [SD] age, 44.6 [18.9] years; 6725 [67.3%] men) to 34 570 prefracture control participants (mean [SD] age, 40.0 [20.5] years; 21 666 [62.7%] men) (Table 1; eFigure in the Supplement). The final sample included 40 438 person-years of patient income for the fracture group and 126 495 person-years for the control group. In the year before injury, the median (interquartile range) individual income of tax filers was $16 847 ($0 to $52 221), and mean (SD) earnings were $38 501 ($84 551).

**Table 1.** Patient Characteristics

Characteristic	Patients, No. (%)		Standardized difference
	Fracture (n = 9997)	Control (n = 34 570)	
Age, mean (SD), y	44.6 (18.9)	40.0 (20.5)^a^	0.23
Men	6725 (67.3)	21 666 (62.7)	0.10
Race/ethnicity			
White	6686 (66.9)	22 654 (65.5)	0.15
African American	2626 (26.3)	9660 (27.9)	
Hispanic	141 (1.4)	128 (0.4)	
Other	544 (5.4)	2128 (6.2)	
Mechanism of injury			
Motor vehicle accident	6046 (60.5)	18 358 (53.1)^b^	0.182
Fall	2563 (25.6)	11 259 (32.6)^b^	
Firearm	446 (4.5)	1732 (5.0)^b^	
Struck	292 (2.9)	910 (2.6)^b^	
Cyclist	266 (2.7)	636 (1.8)^b^	
Machinery	156 (1.6)	783 (2.3)^b^	
Other	228 (2.3)	92 (2.6)^b^	
AIS score, mean (SD)			
Lower extremity	1.9 (1.1)	1.7 (1.1)^b^	0.12
Upper extremity	1.0 (1.1)	0.9 (1.0)^b^	0.13
Abdomen	0.4 (0.9)	0.4 (0.9)^b^	0.04
Face	0.4 (0.6)	0.3 (0.6)^b^	0.11
Head	0.7 (1.0)	0.6 (1.0)^b^	0.14
Neck	0.1 (0.4)	0.1 (0.4)^b^	0.02
Spine	0.4 (0.8)	0.4 (0.8)^b^	0.05
Thorax	0.7 (1.2)	0.7 (1.1)^b^	0.04
Comorbidities			
Alcohol dependence	914 (9.1)	1871 (5.4)^c^	0.14
Depression	665 (6.7)	2471 (7.1)^c^	0.02
Diabetes	911 (9.1)	3931 (11.4)^c^	0.08
Drug use			
IV	162 (1.6)	625 (1.8)^c^	0.01
Non-IV	788 (7.9)	2590 (7.5)^c^	0.02
Hypertension	2292 (22.9)	9798 (28.3)^c^	0.12
Smoker	3049 (30.5)	9859 (28.5)^c^	0.04
Fracture location			
Humerus, clavicle, or scapula	1259 (12.6)	5024 (14.5)^b^	0.06
Radius or ulna	2392 (23.9)	7628 (22.1)^b^	0.04
Femur	2347 (23.5)	10 178 (29.5)^b^	0.14
Tibia or fibula	3653 (36.5)	11 778 (34.1)^b^	0.05
Pelvis or acetabulum	1776 (17.8)	4047 (11.8)^b^	0.17
Hand	1031 (10.3)	3451 (10.0)^b^	0.01
Foot	869 (8.7)	3299 (9.5)^b^	0.03
Open fracture	2887 (28.9)	10 269 (29.7)^b^	0.02
No. of surgical procedures, median (IQR)	4.0 (5.1)	4.5 (6.0)^b^	0.10
Health insurance status			
Private employer-based	3618 (36.2)	9306 (26.9)^d^	0.23
Medicare	2011 (20.1)	7691 (22.2)^d^	
Medicaid	1789 (17.9)	6942 (20.1)^d^	
Uninsured	1264 (12.6)	4877 (14.1)^d^	
Direct purchase	975 (9.8)	3916 (11.3)^d^	
Other public	193 (1.9)	1460 (4.2)^d^	
Tricare/VA/Champ-VA	147 (1.5)	378 (1.1)^d^	

Abbreviations: AIS, Abbreviated Injury Scale; IQR, interquartile range; VA, Veterans Affairs

^a^ Age for the control group is the age of the patient at the time of the match with the fracture group.

^b^ Characteristics of the future fracture for the control group patient.

^c^ Comorbidities present at the time of patient admission for fracture treatment may not have been present at the time of the match with the fracture group.

^d^ Health insurance status at the time of patient admission for fracture treatment may differ at the time of the match with the fracture group.

### Individual Income

**Figure 1.**
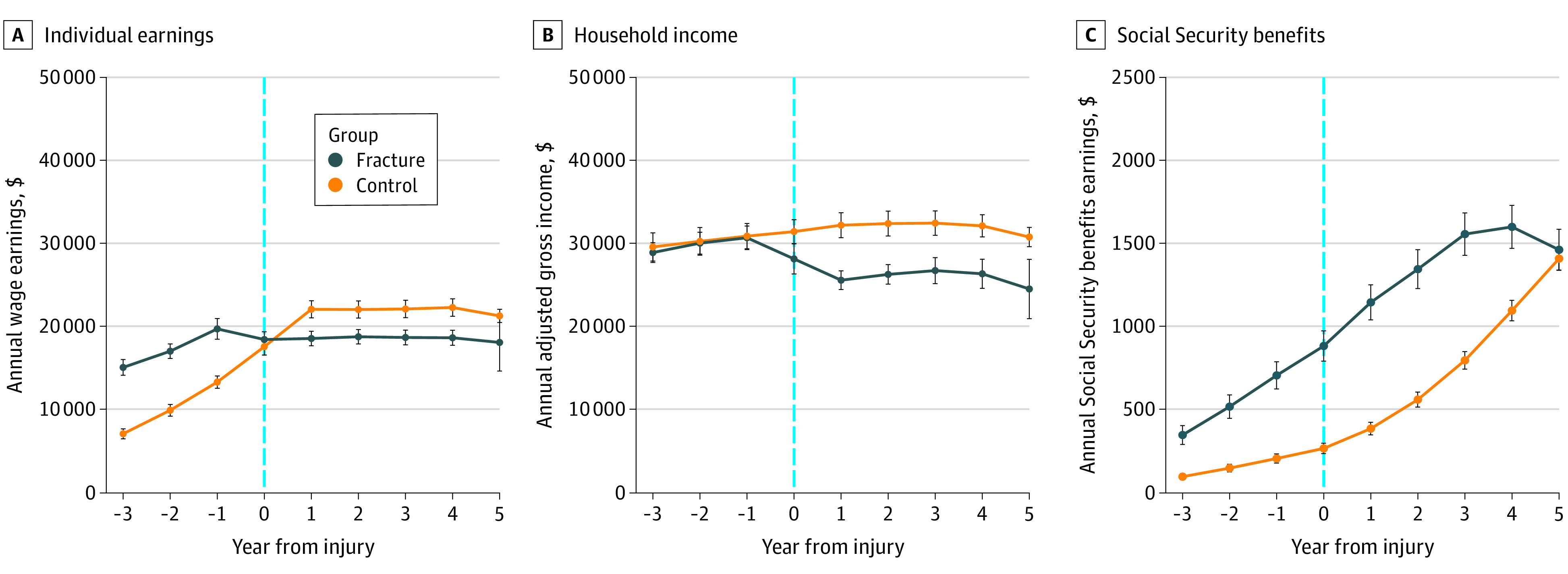
Annual Individual Earnings, Household Income, and Social Security Benefits From 3 Years Prior to Injury to 5 Years After Injury

The mean 5-year reduction in annual individual earnings associated with a fracture was $9865 (95% CI, −$10 686 to −$8862; *P* < .001) (Table 2, Figure 1). Fracture patients lost 81% (95% CI, −82% to −80%; *P* < .001) of counterfactual earnings growth in 5 years postinjury. The $9000 difference in adjusted annual individual earnings loss associated with the fracture remained consistent from 1 to 5 years after injury.

**Table 2.** Difference-in-Difference in Individual Earnings, Household Income, and Social Security Benefits^a^

Outcome	Years from injury	Mean (SD), $		Adjusted difference (95% CI)		*P* value
		Fracture group	Control group	Annual, $	Relative, %	
Individual earnings	Year prior to injury	19 730 (63 510)	13 328 (63 818)	NA	NA	NA
	1	18 575 (44 520)	22 089 (89 517)	−8492 (−9620 to −7364)	−70 (−72 to −68)	<.001
	2	18 784 (44 068)	22 061 (89 315)	−9142 (−10 178 to −8106)	−75 (−77 to −73)	<.001
	3	18 699 (45 164)	22 128 (90 724)	−9505 (−10 500 to −8510)	−77 (−79 to −76)	<.001
	4	18 661 (46 191)	22 304 (90 776)	−9766 (−10 724 to −8807)	−79 (−80 to −78)	<.001
	5	18 100 (175 519)	21 297 (68 643)	−9865 (−10 686 to −8862)	−81 (−82 to −80)	<.001
Household income	Year prior to injury	30 704 (72 301)	30 899 (124 716)	NA	NA	NA
	1	25 599 (57 702)	32 213 (129 712)	−4586 (−5909 to −3263)	−45 (−58 to −42)	<.001
	2	26 299 (59 860)	32 414 (129 335)	−4974 (−6134 to −3814)	−51 (−54 to −49)	<.001
	3	26 756 (79 716)	32 467 (127 137)	−5067 (−6157 to −3977)	−56 (−58 to −54)	<.001
	4	26 370 (89 419)	32 139 (115 073)	−5135 (−6172 to −4098)	−59 (−61 to −57)	<.001
	5	24 545 (182 042)	30 792 (100 422)	−5259 (−6337 to −4181)	−64 (−65 to −62)	<.001
Social Security benefits	Year prior to injury	707 (4166)	207 (2361)	NA	NA	NA
	1	1146 (5373)	387 (3273)	314 (259 to 369)	17.4 (14.7 to 20.1)	<.001
	2	1346 (5957)	562 (3890)	347 (292 to 402)	17.9 (15.4 to 20.6)	<.001
	3	1557 (6508)	798 (4565)	356 (301 to 411)	17.1 (14.6 to 19.7)	<.001
	4	1600 (6592)	1097 (5324)	311 (254 to 368)	14.0 (11.4 to 16.6)	<.001
	5	1462 (6307)	1410 (5972)	206 (147 to 265)	8.0 (5.5 to 10.6)	<.001

Abbreviation: NA, not applicable.

^a^ Adjusted annual difference and adjusted relative difference are the difference-in-difference estimates from the year of injury to the year indicated.

### Household Income

Fractures were associated with a $5259 reduction (95% CI, −$6337 to −$4181; *P* < .001) in annual household income over 5 years postinjury (Table 2, Figure 1). Fracture patient households suffered a 64% reduction (95% CI, −65% to −62%; *P* < .001) in counterfactual income growth after injury. The magnitude of the absolute and relative income loss increased slightly from 1 to 5 years postinjury.

### Social Security Benefits

Fractures were associated with an increase in mean annual Social Security benefits in all 5 time periods (Table 2, Figure 1). The mean difference in annual Social Security benefits compared with preinjury benefits was most substantial 3 years after injury (difference, $356; 95% CI, $301 to $411; *P* < .001). The relative increase in Social Security benefits received postfracture compared with preinjury benefits was consistent, at approximately 17% higher, in years 1 to 3. However, the relative annual difference in Social Security benefits received postfracture compared with prefracture attenuated to 14.0% (95% CI, 11.4% to 16.6%) in the 4 years after injury, and 8.0% (95% CI, 5.5% to 10.6%) in the 5 years postinjury.

### Catastrophic Loss in Individual Income

Fractures increased the risk of catastrophic wage loss 2 years after injury by 11.6% (95% CI, 10.5% to 12.7%; *P* < .001) (eTable 2 in the Supplement). The risk of catastrophic wage loss attributable to a fracture remained similar when we expanded the catastrophic wage loss window from 2 to 5 years after injury (adjusted difference, 11.9%; 95% CI, 10.8% to 13.0%; *P* < .001). Nearly 1 in 10 patients treated for fractures had a 75% or more decline in individual income in the 2 years after injury.

### Heterogeneity of Treatment Effects

**Figure 2.**
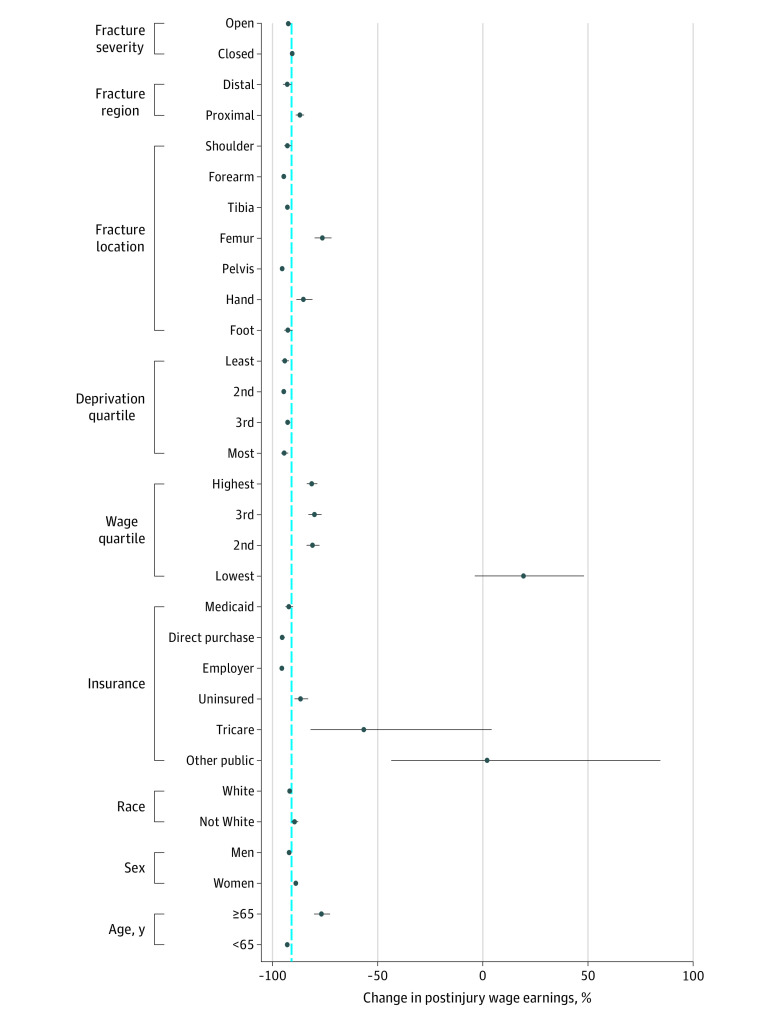
Relative Difference From Counterfactual Individual Earnings Associated With a Fracture The blue dotted line indicates the mean percent change in postinjury wage earnings for the overall sample.

We observed significant heterogeneity in postfracture individual income loss based on patient age, health insurance status, preinjury income, fracture region, and fracture location (Figure 2). Patients with fractures aged 65 years or older sustained less relative income loss (−77%; 95% CI, −80% to −73%) than fracture patients under 65 years of age (−93%; 95% CI, −94% to −92%). We did not observe relative income loss for patients in the lowest income quartile (19%; 95% CI, −4% to 48%). Patients with distal fractures (−93%; 95% CI, −94% to −92%) had greater relative income loss than patients with proximal fractures (−87%; 95% CI, −88% to −86%). The magnitude of relative income loss was marginally lower for patients with hand fractures (−85%; 95% CI, −89% to −81%) or femur fractures (−76%; 95% CI, −80% to −72%) compared with other patients.

**Figure 3.**
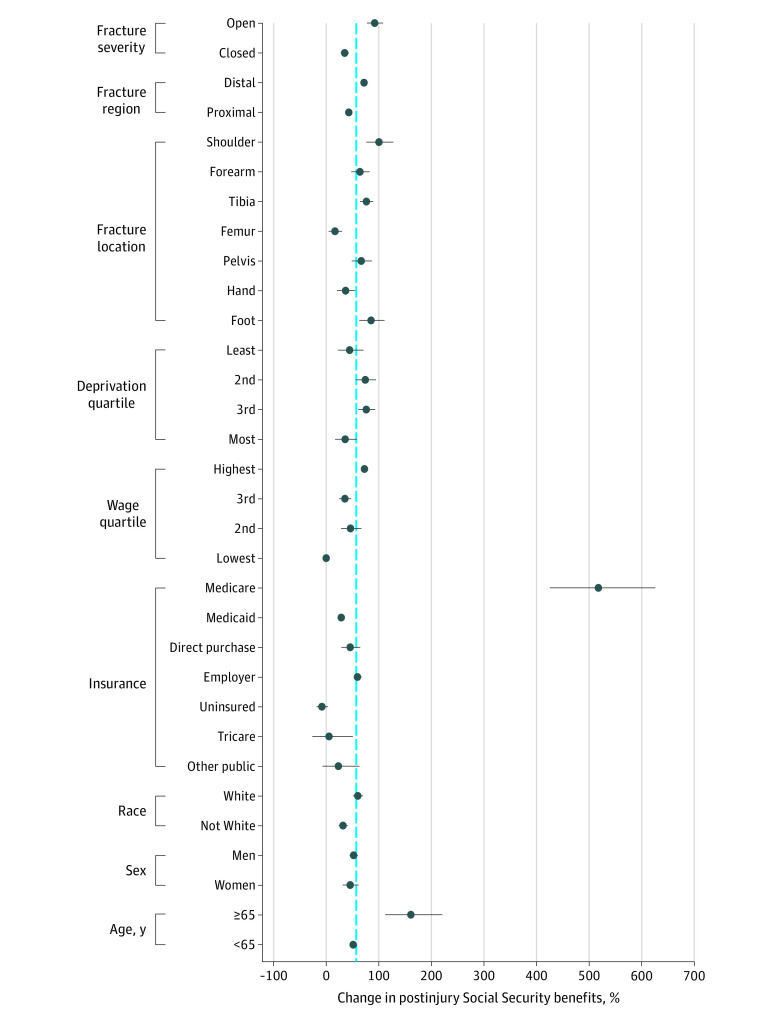
Relative Difference From Counterfactual Social Security Benefits Associated With a Fracture The blue dotted line indicates the mean percent change in postinjury Social Security benefits for the overall sample.

Medicare beneficiaries were significantly more likely to receive increased Social Security benefits (518%; 95% CI, 426% to 626%) than patients with other health insurance coverage (Figure 3). Patients with fractures aged 65 years or older had a greater increase in Social Security benefits (161%; 95% CI, 112% to 221%) than patients under the age of 65 years (51%; 95% CI, 46% to 57%). Patients living in neighborhoods grouped in the middle quartiles of deprivation of our sample had greater increases in Social Security benefits (2nd quartile, 74%; 95% CI, 56% to 95%; 3rd quartile, 76%; 95% CI, 61% to 93%) than the changes observed for patients living in neighborhoods with the least deprivation (45%; 95% CI, 29% to 65%) and most deprivation (36%; 95% CI, 17% to 58%). Patients with distal fractures (72%; 95% CI, 68% to 74%) had a greater relative increase in Social Security benefits than patients with proximal fractures (43%; 95% CI, 40% to 46%). Of the fracture types, patients with femur fractures (17%; 95% CI, 5% to 30%) and patients with hand fractures (37%; 95% CI, 21% to 55%) had the smallest gains in Social Security benefits.

## Discussion

In this study, we used newly available state tax data to obtain precise and comprehensive estimates of an orthopedic injury’s impact on patient income. Fractures were associated with a $9000 decline in annual individual earnings and a $5000 reduction in yearly household income. The mean annual loss in earnings remained consistent for at least 5 years after injury. Nearly 1 in 5 patients had a 50% loss in individual earnings within 2 years of their injury. Fractures were associated with a 17% increase in Social Security benefits in the 3 years after injury, but benefits diminished in years 4 and 5. Patients in the highest income quartile experienced a greater relative earnings loss than patients with lower preinjury earnings, yet received more social welfare.

### Substantial Income Loss After Injury

Our analyses yielded 4 major conclusions. First, the loss in earnings after injury was substantial and persisted for at least 5 years after injury. Nearly half of the sample sustained their injury during the ages of peak income growth. While the decline did not reduce earnings below preinjury levels, early wage stagnation was observed. Our estimated magnitude and duration of relative earnings loss was consistent with previous research on the economic effects of hospital admissions and physical impairment.^25,26^ However, the persistent income loss observed 5 years after injury has not been previously reported in the fracture literature. Understanding the expected duration of income loss is important for guiding clinical care and designing health and social policies.

The loss in household earnings was less than the individual income loss. This suggests a natural smoothing effect where other household members increase their workforce participation to offset the patient’s income decline. Increased income from workers’ compensation or legal settlements, which were included in estimates of household income but not individual income, may also explain part of the observed discrepancy. Financial hardships are often associated with emotional and psychological distress, are an impediment to supporting dependents, and may possibly lead to intergenerational economic effects.^14,27^

### Limited Access to Social Welfare

Our second conclusion was that Social Security benefits significantly increased after the injury but offset less than 10% of lost income in any given year. Similar levels of Social Security protection were reported in a California-based study of hospitalized patients.^27^ Over one-quarter of tax-filing patients treated for fractures reported individual incomes of $0. Lacking formal employment precludes the contributary requirements of many social insurance benefits in the US, such as sick leave, unemployment insurance, and long-term disability benefits. By contrast, research from Denmark suggests that various social programs insured almost 50% of posthospitalization earnings decline.^28^

The US has weaker employment protections than other Organization of Economic Cooperation and Development (OECD) countries,^29^ and is only 1 of 3 OECD countries that do not provide universal access to paid sick leave.^30^ A lack of such social insurance policies likely contributes to the significant financial loss observed after injury. Employer-based health insurance was the most common form of coverage in the study population. Physical impairment that prevents patients from returning to work exposes individuals to further liability for downstream health care expenses. Increasing employment protections for sudden health events, passing legislation for mandatory sick pay benefits, and expanding social welfare programs may reduce the financial implications of orthopedic injury.

### Catastrophic Income Loss

Our third conclusion was that fractures were associated with an 11.6% increase in the risk of catastrophic wage loss in the 2 years following injury. One in 10 patients treated for fractures experienced catastrophic wage loss under a more restrictive definition of a 75% reduction in income. Our estimates of catastrophic wage loss observed in the control group were similar to previous national estimates.^13^ Dobkin et al^26^ estimated an 11% decline in the probability of being employed 3 years after hospitalization, which was consistent with these findings.

### Variations in Income Loss and Social Welfare Benefits

Our fourth conclusion was that preinjury income, age, neighborhood deprivation, fracture region, and the location of injury modified the financial implications of injury. Although the subgroup analysis failed to identify characteristics associated with greater income loss, we observed several factors protective against income loss. With low-income quartile patients reporting no formal preinjury income, it was mathematically impossible for those individuals to sustain income loss. The overall estimates were driven by income losses of individuals from quartiles 2 through 4. Patients aged 65 years or older sustained less relative income loss than patients under 65 years of age. This difference likely results from older patients receiving a higher proportion of their income from fixed, passive sources.^31^ Patients with fractures of the hand and femur were also associated with less relative income loss. The reduced income loss due to a hand fracture may be explained by shorter healing times for that fracture type, the importance of preserved mobility for resuming work, and the ability to perform many work tasks with a fractured hand. Of the included fracture types, femur fractures had the highest correlation with those over 65 years of age.

Access to social welfare after injury varied. The increase in social spending on older adults compared with working-age adults has been previously reported.^32^ This research has shown a U-shaped association between neighborhood deprivation and Social Security benefits. Specifically, patients living in the least and most deprived neighborhoods received significantly less benefits than patients residing in the mid-level deprivation quartiles. These differences may reflect coverage gaps or variations in benefit demands. Medicare beneficiaries and elderly patients had a considerable change in Social Security benefits. A 2018 study^26^ reported a similar finding attributed to Social Security Retirement Income eligibility and the higher upper limit for retirement benefits compared with other Social Security benefits. Open fractures, a common marker of more severe injury, had a significant increase in Social Security benefits compared with less severe fractures. Patients with femur and hand fractures received less Social Security benefits than other fracture types, which plausibly can be associated with less relative income loss.

### Limitations

This study had several limitations. The primary outcome was obtained from W-2 reported data, which does not include self-employment income, tips, or earnings from jobs that paid less than $1800 a year, all likely important sources of earnings for this population. Certain covariates previously associated with economic loss after injury, such as occupation, were not available within our data. Although the proportion of nonfilers in our sample was similar to previous studies and IRS reporting, we cannot completely discount the endogenous effects of nonfiling.^15,16,33^ We do not have specific data on workers’ compensation or legal compensation received by the patients. In most cases, these sources of income would be included as federal adjusted gross income that comprises our household income endpoint. Our heterogeneity of treatment effect analysis did not control for family-wise type I error, and should be considered exploratory, not confirmatory. Given the potential challenges of heterogeneity of treatment effect analysis in observational data leading to false positive and false negative conclusions, we adopted a descriptive approach. Despite these limitations, we believe the administrative data used in the study is a profound improvement over the self-reported data used in previous studies. Finally, the study sample was obtained from a single trauma center, and tax data was specific to 1 state, which enhances internal validity but may limit the generalizability of the findings to other hospitals and states.

## Conclusions

The findings of this study suggest that fractures significantly reduced patient earnings for up to 5 years after injury. Gains in social welfare payments were limited, covering less than 10% of annual income losses. Patients aged 18 to 65 years and patients with preinjury incomes in the upper quartiles suffered the greatest loss in earnings. Increases in Social Security benefits were most profound in patients older than 65 years at the time of injury.

## Notes

Supplement. **eTable 1.** Parallel Trends Test for Pre-Injury Treatment Group Comparisons **eTable 2.** Risk of Catastrophic Wage Loss After Injury **eFigure.** Patient Selection

## References

[1] Bhashyam AR, McGovern MM, Mueller T, Heng M, Harris MB, Weaver MJ The personal financial burden associated with musculoskeletal trauma. J Bone Joint Surg Am. 2019;101(14):1245-1252. doi:10.2106/JBJS.18.01114 31318803

[2] O’Hara NN, Isaac M, Slobogean GP, Klazinga NS The socioeconomic impact of orthopaedic trauma: a systematic review and meta-analysis. PLoS One. 2020;15(1):e0227907. doi:10.1371/journal.pone.0227907 31940334 PMC6961943

[3] Morrison ER, Gupta A, Olson L, Cook L, Keenan H Health and financial fragility: evidence from car crashes and consumer bankruptcy. University of Chicago Coase-Sandor Institute for Law & Econ Research Paper No. 655. Published November 13, 2013. doi:10.2139/ssrn.2353328

[4] Sullivan D, von Wachter T Job displacement and mortality: an analysis using administrative data. Q J Econ. 2009;124(3):1265-1306. doi:10.1162/qjec.2009.124.3.1265

[5] Pool LR, Burgard SA, Needham BL, Elliott MR, Langa KM, Mendes de Leon CF Association of a negative wealth shock with all-cause mortality in middle-aged and older adults in the United States. JAMA. 2018;319(13):1341-1350. doi:10.1001/jama.2018.2055 29614178 PMC5933380

[6] Himmelstein DU, Warren E, Thorne D, Woolhandler S Illness and injury as contributors to bankruptcy. Health Aff (Millwood). 2005;(Suppl Web Exclusives):W5-63-W5-73. doi:10.1377/hlthaff.W5.63 15689369

[7] Himmelstein DU, Thorne D, Warren E, Woolhandler S Medical bankruptcy in the United States, 2007: results of a national study. Am J Med. 2009;122(8):741-746. doi:10.1016/j.amjmed.2009.04.012 19501347

[8] Stephan K, Huber S, Häberle S, ; TraumaRegister DGU Spinal cord injury—incidence, prognosis, and outcome: an analysis of the TraumaRegister DGU. Spine J. 2015;15(9):1994-2001. doi:10.1016/j.spinee.2015.04.041 25939671

[9] Savitsky B, Givon A, Rozenfeld M, Radomislensky I, Peleg K Traumatic brain injury: it is all about definition. Brain Inj. 2016;30(10):1194-1200. doi:10.1080/02699052.2016.1187290 27466967

[10] Dimick JB, Ryan AM Methods for evaluating changes in health care policy: the difference-in-differences approach. JAMA. 2014;312(22):2401-2402. doi:10.1001/jama.2014.16153 25490331

[11] MacKenzie EJ, Bosse MJ, Kellam JF, Characterization of patients with high-energy lower extremity trauma. J Orthop Trauma. 2000;14(7):455-466. doi:10.1097/00005131-200009000-00001 11083607

[12] Court-Brown CM, Aitken SA, Duckworth AD, Clement ND, McQueen MM The relationship between social deprivation and the incidence of adult fractures. J Bone Joint Surg Am. 2013;95(6):e321-e327. doi:10.2106/JBJS.K.00631 23515993

[13] Dahl M, DeLeire T, Schwabish JA Estimates of year-to-year volatility in earnings and in household incomes from administrative, survey, and matched data. J Human Resources. 2011;46(4):750-774. doi:10.1353/jhr.2011.0000

[14] Mejía ST, Settersten RA Jr, Odden MC, Hooker K Responses to financial loss during the great recession: an examination of sense of control in late midlife. J Gerontol B Psychol Sci Soc Sci. 2016;71(4):734-744. doi:10.1093/geronb/gbv054 26307482 PMC5013892

[15] Chetty R, Hendren N, Katz LF The effects of exposure to better neighborhoods on children: new evidence from the moving to opportunity experiment. Am Econ Rev. 2016;106(4):855-902. doi:10.1257/aer.20150572 29546974

[16] Chetty R, Hendren N, Kline P, Saez E Where is the land of opportunity? the geography of intergenerational mobility in the United States. Q J Econ. 2014;129(4):1553-1623. doi:10.1093/qje/qju022

[17] Chetty R, Stepner M, Abraham S, The association between income and life expectancy in the United States, 2001-2014. JAMA. 2016;315(16):1750-1766. doi:10.1001/jama.2016.4226 27063997 PMC4866586

[18] Kind AJH, Jencks S, Brock J, Neighborhood socioeconomic disadvantage and 30-day rehospitalization: a retrospective cohort study. Ann Intern Med. 2014;161(11):765-774. doi:10.7326/M13-2946 25437404 PMC4251560

[19] Kind AJH, Buckingham WR Making neighborhood-disadvantage metrics accessible—the neighborhood atlas. N Engl J Med. 2018;378(26):2456-2458. doi:10.1056/NEJMp1802313 29949490 PMC6051533

[20] Bilinski A, Hatfield LA Seeking evidence of absence: reconsidering tests of model assumptions. Preprint. Posted online May 8, 2018 Accessed December 17, 2020. arXiv 180503273. https://arxiv.org/abs/1805.03273v1

[21] Mishel L, Gould E, Bivens J Wage stagnation in nine charts. Econ Policy Inst. January 2015:2-13. Accessed January 21, 2021. https://www.epi.org/publication/charting-wage-stagnation/

[22] US Bureau of Labor Statistics Labor Force Statistics from the Current Population Survey. Updated October 23, 2020 Accessed June 17, 2020. https://www.bls.gov/cps/earnings.htm

[23] Wooldridge JM Introductory Econometrics: A Modern Approach. Cengage Learning; 2013.

[24] Rubin DB Inference and missing data. Biometrika. 1976;63(3):581-592. doi:10.1093/biomet/63.3.581

[25] Charles KK The longitudinal structure of earnings losses among work-limited disabled workers. J Human Resources. 2003;38(3):618-646. doi:10.2307/1558770

[26] Dobkin C, Finkelstein A, Kluender R, Notowidigdo MJ The economic consequences of hospital admissions. Am Econ Rev. 2018;108(2):308-352. doi:10.1257/aer.20161038 30091560

[27] Kyle MA, Blendon RJ, Benson JM, Abrams MK, Schneider EC Financial hardships of Medicare beneficiaries with serious illness. Health Aff (Millwood). 2019;38(11):1801-1806. doi:10.1377/hlthaff.2019.00362 31682505

[28] Fadlon I, Nielsen TH Household responses to severe health shocks and the design of social insurance. NBER Work Pap Ser. National Bureau of Economic Research; 2015. NBER publication 21352. Accessed December 17, 2020. doi:10.3386/w21352

[29] Organization for Economic Cooperation and Development OECD Employment Outlook 2018. OECD; 2018. doi:10.1787/empl_outlook-2018-en

[30] Maclean JC, Pichler S, Ziebarth NR Mandated sick pay: coverage, utilization, and welfare effects. NBER Work Pap Ser. National Bureau of Economic Research; 2020. NBER publication 26832. Accessed December 17, 2020. doi:10.3386/w26832

[31] Wu KB Sources of income for older Americans, 2012. AARP Public Policy Institute; 2013.

[32] Tikkanen RS, Schneider EC Social spending to improve population health—does the United States spend as wisely as other countries? N Engl J Med. 2020;382(10):885-887. doi:10.1056/NEJMp1916585 32130810

[33] Fullerton D, Rao NL The lifecycle of the 47 percent. Natl Tax J. 2019;72(2):359-396. doi:10.17310/ntj.2019.2.03

