# Supplementary material for: Analysis of Patient Income in the 5 Years Following a Fracture Treated Surgically
Source: JAMA Netw Open. 2021 Feb 8;4(2):e2034898. doi: 10.1001/jamanetworkopen.2020.34898 (PMC7871192; doi:10.1001/jamanetworkopen.2020.34898)
Supplement: Supplement. — eTable 1. Parallel Trends Test for Pre-Injury Treatment Group Comparisons eTable 2. Risk of Catastrophic Wage Loss After Injury eFigure. Patient Selection [file jamanetwopen-e2034898-s001.pdf]

Supplemental Online Content

O'Hara NN, Slobogean GP, Klazinga NS, Kringos DS. Analysis of patient income in the 5 years following a fracture treated surgically. *JAMA Netw Open*. 2021;4(2):e2034898. doi:10.1001/jamanetworkopen.2020.34898

eTable 1. Parallel Trends Test for Pre-Injury Treatment Group Comparisons
eTable 2. Risk of Catastrophic Wage Loss After Injury
eFigure. Patient Selection

This supplemental material has been provided by the authors to give readers additional information about their work.

© 2021 O'Hara NN et al. *JAMA Network Open.*

eTable 1. Parallel Trends Test for Pre-Injury Treatment Group Comparisons[a]

	**Annual Mean Difference (SE)**	**Value**	**Equivalence Margin**	**Equivalence *P* Value**
Individual Earnings	-$792 (469)	0.09	$2000	0.005
Household Income	$241 (1032)	0.82	$2000	0.044
Social Security Benefits	$125 (20)	<0.01	$200	<0.001

[a] Adjusted for patient age and year of injury.

© 2021 O'Hara NN et al. *JAMA Network Open.*

eTable 2. Risk of Catastrophic Wage Loss After Injury

**Level of Catastrophic Wage Loss**	**Years Post-Injury**	**Fracture (n = 9997)**	**Control (n = 28 785)**	**Adjusted Mean Difference (95% CI)**	***P* Value**
25%	2 years	2989 (29.9%)	3857 (13.4%)	18.7% (17.3%–20.1%)	<0.001
	5 years	2899 (29.0%)	4318 (15.0%)	17.6% (16.3%–18.9%)	<0.001
**50%**	**2 years**	**1789 (17.9%)**	**2245 (7.8%)**	**11.6% (10.5%–12.7%)**	**<0.001**
	5 years	1809 (18.1%)	2418 (8.4%)	11.9% (10.8%–13.0%)	<0.001
75%	2 years	950 (9.5%)	1267 (4.4%)	6.0% (5.1%–6.8%)	<0.001
	5 years	1000 (10.0%)	1267 (4.4%)	6.8% (5.9%–7.6%)	<0.001

© 2021 O'Hara NN et al. *JAMA Network Open.*

eFigure. Patient Selection

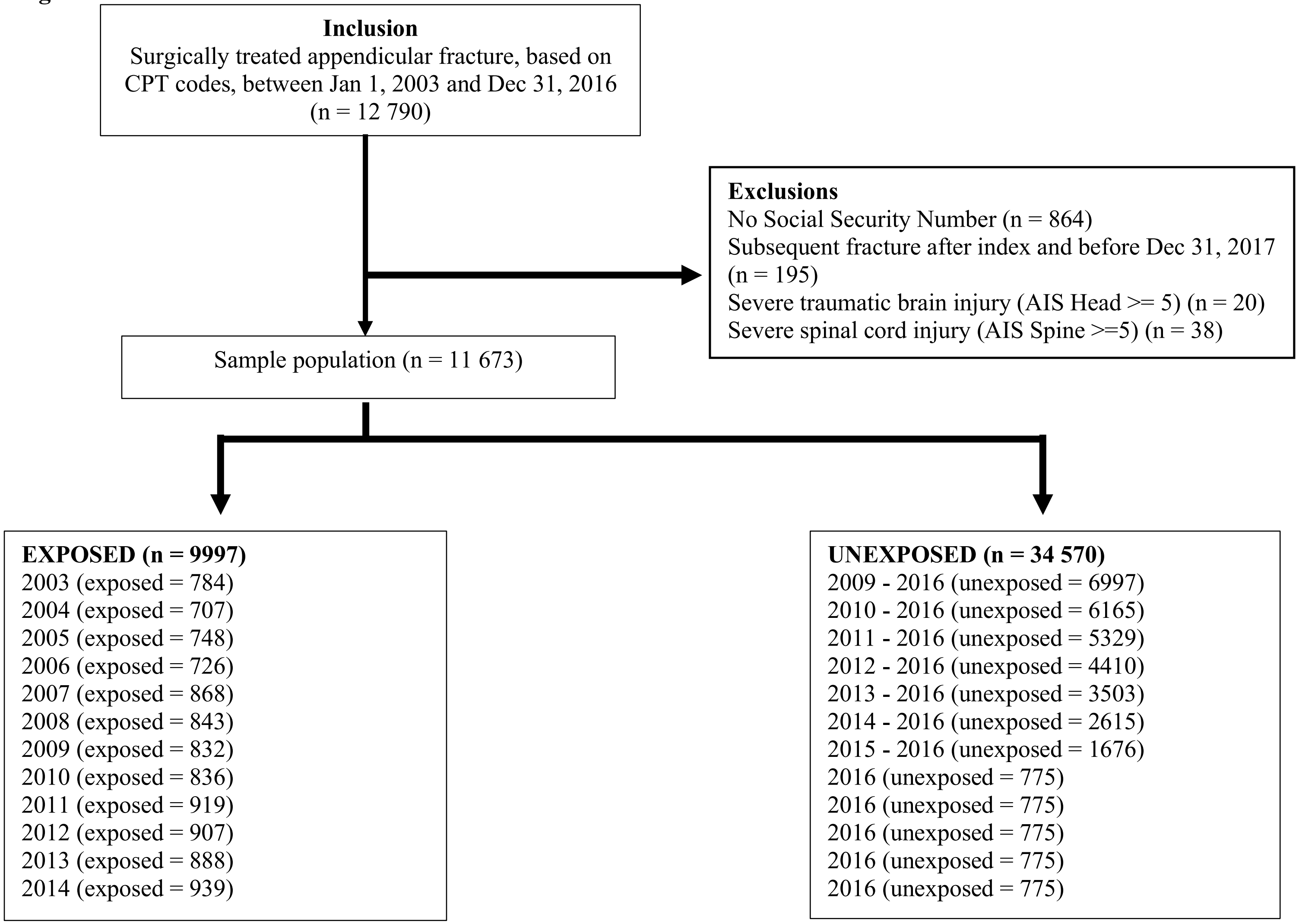

© 2021 O'Hara NN et al. *JAMA Network Open.*
